# Western Medico-Chirurgical Journal

**Published:** 1851-09

**Authors:** 


### Part 3.—Editorial.

ARTICLE I.

### WESTERN MEDICO-CHIRURGICAL JOURNAL.

This periodical is the organ of the Medical Department of the Iowa University. That institution is a prolongation of the Medical School that attempted to set up in St. Charles, Ill., a number of years ago; went from there to Laporte, Ind.; its organizers left there for *cause*, and set up the School in Rock Island, Ill.; thence removed it to Davenport, Iowa; and last of all moved it to the city of Keokuk.

As most of our readers know the dishonorable course pursued by this heretofore moving, trading, secret-remedy-promulging establishment toward Rush Medical College, we had not deemed it advisable to notice it in the Journal. But since the organ of the School has been publishing misrepresentations and false accusations against Rush Medical College, and has the audacity to say that those connected with that School do not deny them, we hope our readers will pardon us for thus briefly alluding to it.

As to Rush Medical College, its course has uniformly been open; its history is known to the profession at large. Its position upon the subject of fees, the alleged cause of offence, was assumed after it had been publicly advocated by Dr. Stevens, the eminent Ex-President of the American Medical Association, and adopted by the University of Michigan, one of its nearest neighbors. It stands in that position from principle, believing it to be the true policy; and its faculty will rejoice at the time when all the Schools of the country shall come up to it, regarding it as the harbinger of better times for the profession.

